# The Equity Tool for Valuing Global Health Partnerships

**DOI:** 10.9745/GHSP-D-21-00316

**Published:** 2022-04-28

**Authors:** Charles P. Larson, Katrina M. Plamondon, Leslie Dubent, Frank Bicaba, Abel Bicaba, Tran Hung Minh, An Nguyen, Jacques E. Girard, Jean Ramdé, Theresa W. Gyorkos

**Affiliations:** a Canadian Association for Global Health (formerly Canadian Coalition for Global Health Research), Ottawa, Canada.; b Department of Epidemiology, Biostatistics and Occupational Health, School of Population and Global Health, Faculty of Medicine and Health Sciences, McGill University, Montréal, Canada.; c School of Nursing, Faculty of Health and Social Development, University of British Columbia, Okanagan, Canada.; d Société d'Études et de Recherche en Santé Publique, Ouagadougou, Burkina Faso.; e Center for Creative Initiatives in Health and Population, Hanoi, Vietnam.; f HealthBridge Vietnam, Hanoi, Vietnam.; g Direction en Santé Mondiale, Faculté de Médecine, Université Laval, Québec, Canada.

## Abstract

Global health partnerships (GHPs) involve complex relationships between individuals and organizations, often joining partners from high-income and low- or middle-income countries around work that is carried out in the latter. Therefore, GHPs are situated in the context of global inequities and their underlying sociopolitical and historical causes, such as colonization. Equity is a core principle that should guide GHPs from start to end. How equity is embedded and nurtured throughout a partnership has remained a constant challenge. We have developed a user-friendly tool for valuing a GHP throughout its lifespan using an equity lens. The development of the EQT was informed by 5 distinct elements: a scoping review of scientific published peer-reviewed literature; an online survey and follow-up telephone interviews; workshops in Canada, Burkina Faso, and Vietnam; a critical interpretive synthesis; and a content validation exercise. Findings suggest GHPs generate experiences of equity or inequity yet provide little guidance on how to identify and respond to these experiences. The EQT can guide people involved in partnering to consider the equity implications of all their actions, from inception, through implementation and completion of a partnership. When used to guide reflective dialogue with a clear intention to advance equity in and through partnering, this tool offers a new approach to valuing global health partnerships. Global health practitioners, among others, can apply the EQT in their partnerships to learning together about how to cultivate equity in their unique contexts within what is becoming an increasingly diverse, vibrant, and responsive global health community.

## Abstract (precis)

There is a need to more comprehensively identify and respond to equity in global health partnerships. The Equity Tool can support dialogue at any stage of a partnership, by individuals at any level. This assists partnerships to embrace ways of recognizing, understanding, and advancing equity in all their processes.

## Teaser

**Key Findings:**

- There is a need to more comprehensively advance equity in global health partnerships.
- The Equity Tool (EQT) offers a practical guide for considering equity in 4 domains of practice: governance and process, procedures and operations, progress and impacts, and power and inclusion.
- The EQT is equity focused, user friendly, and can support reflective dialogue at any stage of the partnership, by individuals at any level in the partnership.

**Key Implications:**

- The EQT will spark questions that invite people to pause and think about their experiences within a partnership.
- By periodically engaging in relational, reflective dialogue about how equity is experienced in a global health partnership using the EQT, partners can embrace ways of recognizing, understanding, and advancing equity in all their processes.
- The EQT offers prompts for reflective dialogue about how equity or inequity is experienced in many different ways and moments throughout the process of partnering, which require attention to creating safe, learning-focused conversations with clear intentions and respect for the contributions and vulnerability of all involved.

Résumé en français à la fin de l'article.

## INTRODUCTION

Over the past decade, equity has become recognized as a core value guiding the practice of global health. Whether oriented toward research, capacity building, or development, partnerships are often promoted as mechanisms for working in global health, with equity more-or-less centered in the process and practices in global health. Partnerships involve complex relationships between individuals and organizations, each with their particular positions, context, needs, resources, and agendas. In global health, partnerships are common between organizations in high-income countries (HICs) and those in low- and middle-income countries (LMICs). Such partnerships can be difficult to navigate, particularly because issues of power are rooted in complex sociopolitical and economic histories.^1^^,^^2^ GHPs exist in the ambient context of persistent health and economic inequities between HICs and LMICs and continued calls for the decolonizing of global health.^3^ These inequities are caused by the unfair distribution of resources, wealth, and power.^4^^,^^5^ Addressing (and even discussing) equity considerations and issues of power can be both sensitive and difficult, especially if such discourse is viewed as being outside the immediate goals of the partnership. Indeed, global health has had a long history of **not** directly talking about these issues.^3^ Yet, GHPs that consider issues of equity in their processes and structures hold greater potential for lasting health impact and building local capacity than those that do not.^6^^,^^7^ Attempts to construct a meaningful guide on what makes GHPs successful are varied and context-specific, often without clear consideration of issues of equity.^8^^–^^10^

GHPs that consider issues of equity in their processes and structures hold greater potential for lasting health impact and building local capacity than those that do not.

The Canadian Coalition for Global Health Research (CCGHR)* over the past decade has prioritized the promotion of equity in GHPs, resulting in the development of a Partnership Assessment Tool^11^^,^^12^ and the equity-centered Principles for Global Health Research.^13^^,^^14^ Another notable effort to amplify attentiveness to equity in GHPs is the Council on Health Research for Development's Research Fairness Initiative.^15^ These resources point to the importance of equity in partnering processes yet tend to focus on aspirational ideals or higher-level considerations rather than on the day-to-day practices of partnering. Extending the scope of these equity-centered aspirational resources, we sought to develop a complementary, practical, user-friendly tool (the EQT) to support ongoing attention to issues of equity in the day-to-day practices of GHPs. In this article, we present the EQT, briefly describe how it was developed, and provide comprehensive and practical guidance on how it may be used. We invite those involved in GHPs to open a productive and relationship-building dialogue about the complex relational processes that lead to more equity-centered partnerships.

## METHODS

The development of the EQT was informed by 5 distinct inputs: (1) a scoping review of scientific published peer-reviewed literature; (2) an online survey and follow-up telephone interviews with global health practitioners and researchers; (3) workshops in Canada, Burkina Faso, and Vietnam; (4) a critical interpretive synthesis; and (5) a content validation exercise (Supplement 1 includes a detailed description of these inputs).

### Ethics Approval

We obtained ethics approvals from the Institutional Review Board of the Faculty of Medicine at McGill University (IRB # A01-E03-19A) and the University of British Columbia Okanagan (REB#H19-00232-A002). All participants gave their informed consent before participating in the online survey (written consent), telephone interviews (verbal consent), and workshops.

## RESULTS

Consolidating the results from the 5 inputs (Supplement 2), the research team mapped what and how issues of equity were either being assessed or considered in GHPs. Guided by the equity-centered CCGHR Principles for Global Health Research,^13^ data showing specific and promising ways to practice equity were grouped under 4 different domains of practice (governance and process; procedures and operations; progress and impacts; and power and inclusion, [Table]^16^^–^^43^). From each of these promising ways to put practices into action, a set of statements for each domain of practice were derived—each intended to illuminate how people engaged in a GHP feel about the ways that equity is functionally working and experienced by themselves, as an individual, and in the partnership overall.

**TABLE.** Overview of Partnering Practices and Sources of Evidence From the Scoping Review and Critical Interpretive Synthesis

Domains of Practice	Partnering Practices	Promising Ways to Put These Practices Into Action	Sources of Evidence From Scoping Review/ Critical Interpretive Synthesis (First Author)
Governance and process	Practices that have to do with assigning authority, making decisions, and creating accountability as people work together toward an agreed end. Ways in which partnerships set priorities and directions, seek alignment between personal, organizational, and partnership goals; set intentions; and determine who gets to be involved in these processes.	Set shared priorities and objectives	Beran,^16^ Buse,^17^ Citrin,^18^ Dean,^19^ El Bcheraoui,^20^ Herrick,^21^ John,^22^ Kamya,^23^ Leffers,^24^ Lips,^25^ Neuhann^26^, Njelesani,^27^ Pattberg,^28^ Perez-Escamilla,^29^ Yarmoshuk^30^
		Make decisions with transparency	Beran, Bruen,^31^ Buse, Citrin, Coffey,^32^ Herrick, John, Kamya, Perez-Escamilla, Steenhoff,^33^ Storr,^34^ Upvall^35^
		Establish shared values and vision	Beran, Birch,^36^ Buse, Citrin, Coffey, El-Bcheraoui, John, Lipsky, Murphy, Ndenga,^37^ Pattberg, Shriharan,^38^ Underwood,^39^ Yarmoshuk, Yassi^40^
		Articulate needs and expectations of what skills, roles, people are needed	Beran, Herrick, Lipsky, Pattberg, Sandwell
		Establish agreements (e.g., memorandum of understanding)	Beran, Buse, Lipsky, Steenhoff
		Create transparent accountability mechanisms	Bruen, Perez-Escamilla
		Prioritize authentic partnering and reciprocity	Beran, Dean, Kamya, John, Murphy, Neuhann, Njelesani, Ridde,^41^ Sriharan, Storr, Theissen,^42^ Yarmoshuk
		Share leadership, decision making	Beran, Coffey, Dean, Kamya, Lipsky, Neuhann, Pattberg, Steenhoff, Storr, Theissen, Upvall
		Clarify roles and responsibilities	Birch, John, Kamya, Lipsky, Neuhann, Pattberg
		Communicate clearly and often	Beran, Birch, Coffey, Dean, John, Neuhann, Njelesani, Perez-Escamilla, Ridde, Steenhoff, Storr
		Build trust and relationships	Beran, Birch, Buse, Citrin, Coffey, Herrick, John, Kamya, Leffers, Lipsky, Ndenga, Njelesani, Pattberg, Ramaswamy,^43^ Sandwell, Sriharan, Storr, Theissen, Upvall, Yassi
		Plan for sustainable resourcing and finances	Beran, Birch, Buse, Dean, Herrick, John, Leffers, Lipsky, Pattberg, Sandwell, Steenhoff, Yarmoshuk, Yassi
Procedures and operations	Practices that have to do with the day-to-day management and conduct of work by people involved in the partnership. These practices include what routine opportunities people are afforded by virtue of participating in the partnership (e.g., gaining skills) and the day-to-day operational procedures (e.g., budget allocation; how resources are shared).	Use conflict-resolution mechanisms	Bruen, Buse, Lipsky, Neuhann, Pattberg, Perez-Escamilla, Steenhoff
		Distribute resources equitably	Beran, Citrin, Dean, Herrick, Neuhann, Pattberg, Storr, Yarmoshuk
		Provide fair salaries and compensation	Dean, Herrick, Ridde, Yarmoshuk
		Be aware of, and respond to, local needs, cultures, and contexts	Beran, Birch, Citrin, Coffey, John, Leffers, Ramaswamy, Ridde, Sriharan, Storr, Underwood, Upvall, Yarmoshuk
		Actively monitor ethical issues	Birch, Buse, Murphy, Njelesani, Ridde, Yassi
		Use transparent management and evaluation mechanisms	Birch, Bruen, Citrin, El Bcheraoui, Kamya, Lipsky, Njelesani, Neuhann, Pattberg, Ramaswamy, Steenhoff, Yassi
		Do risk assessments and mitigate unpredictable (or unintended) changes, impacts, and risks	Buse, Murphy, Pattberg, Ridde, Steenhoff
		Recognize contributions	Beran, Buse, Kamya, Lipsky
		Make efforts to mitigate inequities in wealth, resources, and power	Dean, Herrick, Murphy, Njelesani, Pattberg, Ridde, Yarmoshuk, Yassi
		Recognize inequities that exist both within the partnership setting and between partners	Birch, Buse, Herrick, Njelesani, Pattberg, Ridde, Storr, Underwood, Upvall, Yarmoshuk
		Adopt adaptive, flexible, responsive implementation approaches	Citrin, Lipsky, Perez-Escamilla, Ramaswamy
		Use evidence to inform action	Buse, El-Bcheraoui, Pattberg
		Provide access to mentoring and training	Birch, Dean, Herrick, John, Leffers, Ridde, Steenhoff, Underwood, Upvall, Yarmoshuk
Progress and impacts	Practices that have to do with determining and setting goals for personal, partner, community, and overall benefits of the partnership and its outputs, outcomes, and products—both actual and potential (e.g., alignment with local priorities), including long-term sustainability of the partnership and/or its benefits.	Monitor performance and impacts	Beran, Bruen, Buse, Dean, Herrick, Leffers, Pattberg, Ramaswamy, Yassi
		Focus on learning and solutions	Citrin, Dean, El-Bcheraoui, John, Neuhann, Njelesani, Ramaswamy, Ridde, Storr, Underwood, Upvall, Yassi
		Plan knowledge translation efforts that respond to local needs	Beran, Birch, Coffey, Murphy, Njelesani
		Consider long-term vision and impacts, including on human rights, environment, Sustainable Developments Goals, how the partnership will advance equity	Birch, Citrin, Coffey, El-Bcheraoui, Herrick, John, Leffers, Lipsky, Njelesani, Pattberg, Perez-Escamilla, Ramaswamy, Steenhoff, Storr, Theissen
		Prioritize positive local impacts	Buse, Citrin, Coffey, Herrick, Lipsky, Neuhann, Pattberg, Ramaswamy, Ridde, Sriharan
		Actively enable people to make meaningful contributions	Beran, Buse, Coffey, Dean, Lipsky, Njelesani, Ridde, Upvall, Yassi
		Consider equity in authorship and publication	Citrin, Dean, Murphy, Ridde
Power and inclusion	Practices that have to do with awareness and responsiveness to power dynamics, issues of equity and representation, voice, feelings of genuine inclusion, and relational experience of being in a partnership.	Practice inclusive, participatory processes that value different perspectives	Coffey, Lipsky, Murphy, Ngenga, Neuhann, Njelesani, Ridde, Sriharan, Theissen, Yarmoshuk, Yassi
		Know and use partners' strengths	Buse, Lipsky, Neuhann, Njelesani, Ramaswamy, Ridde, Sandwell, Theissen, Upvall
		Include diverse perspectives and all the relevant stakeholders, especially across genders and by communities intended as beneficiaries	Beran, Birch, Bruen, Buse, Coffey, Kamya, Leffers, Ridde, Sandwell, Steenhoff, Theissen, Yarmoshuk
		Seek to understand diverse perspectives and their relationship to power, mitigate power imbalances	Beran, Citrin, Coffey, Dean, John, Murphy, Njelesani, Ridde, Sriharan, Storr, Upvall
		Value and recognize technical skills	Beran, Kamya, Lipsky, Njelesani
		Strive for reciprocity	Citrin, Kamya, Lipsky, Njelesani, Ridde, Sandwell, Theissen, Upvall, Yarmoshuk
		Invite genuine participation, listen actively to all relevant stakeholders, avoid token involvement	Beran, Citrin, Dean, Ridde, Sandwell, Sriharan, Storr, Theissen, Yarmoshuk

From each of these promising ways to put equity practices into action, a set of statements were derived that were intended to illuminate how people engaged in a GHP feel about the ways in which equity is functionally working.

The cumulative results from the 5 inputs resulted in a set of 55 statements that form the final EQT (Figure). Because definitions of partnership terms and indicators were rarely defined or used congruently in the literature and to be transparent about the definitions used herein in developing the EQT tool, definitions are included in Supplement 3. The French version of the tool is provided in Supplement 4.

**FIGURE:**
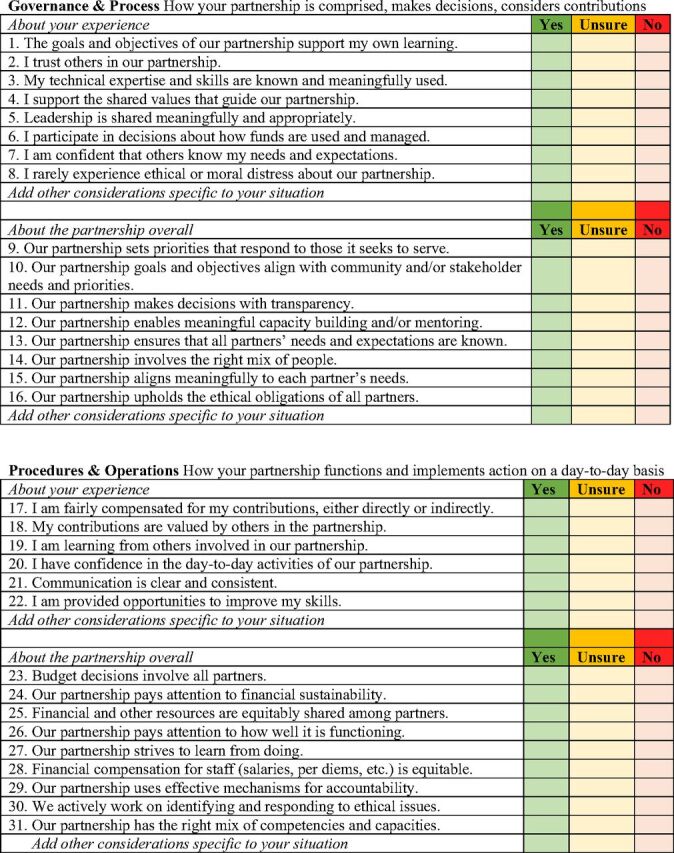

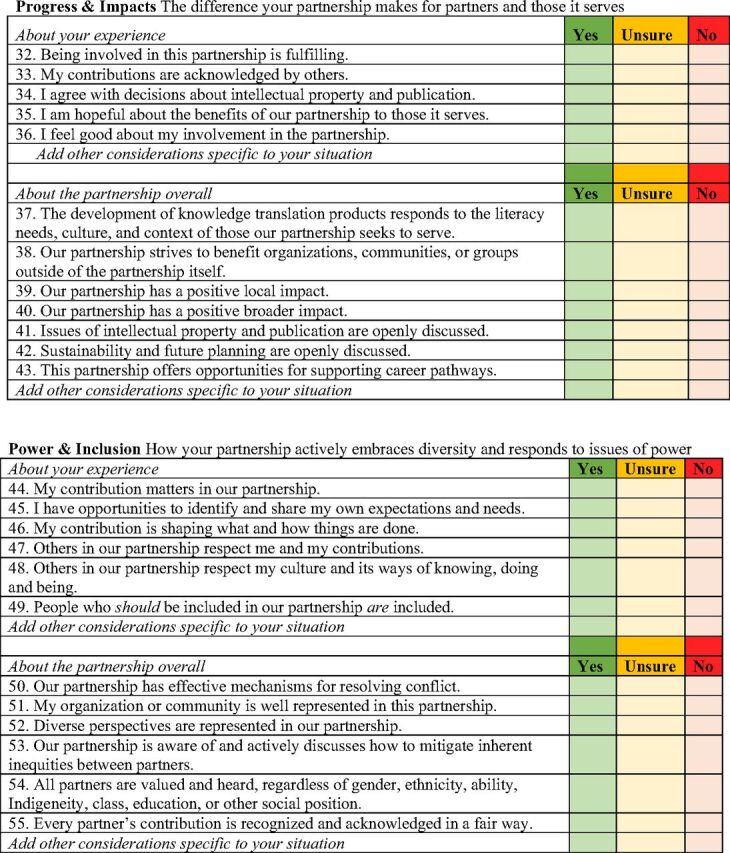
The Equity Focused Tool for Valuing Global Health Partnerships

The primary intent of the EQT is to support dialogue that enables people involved in partnering to reflect on their own experiences and to identify how equity is reflected (or not) in partnering practices or processes. It is important to begin conversations using the EQT with shared intention setting that emphasizes the use of the tool as a mechanism to identify equity considerations and support equity-centered practices, working together to learn from each other about how to advance equity in a good way. The tool will spark questions that allow people to pause and think about their experiences of partnering. Different people involved in the partnership will experience the partnership and equity within it differently. These differences are expected and provide a foundation for exploring how to better understand how some aspect of partnering is (or is not) working to advance equity. Partnerships may find it useful to use the tool to guide dialogue from the earliest phases of partnering. Specific effort to use the dialogue as a resource in identifying how equity considerations can be integrated into the work of the partnership. Partnerships may choose to revisit the EQT periodically and when they end or transform into something new. Pausing to reflect on equity considerations will support more equitable engagement in future partnerships.

Before entering into a GHP, organizations, and staff less familiar with principles of equity and related issues (e.g., cultural humility and issues of power and privilege) need to be considered. Supplement 5 lists several references that can support people to engage in conversations using the EQT in ways that are safe, respectful, and productive.

## DISCUSSION

Using an iterative, mixed-methods approach, our research culminated in creating a tool to guide practical, equity-centered dialogue about how a GHP is functioning. The literature review identified several GHP assessment tools. These tools reflected the authors' interpretation of what contributes to good partnership practices based on their experiences in GHPs that were created to support capacity building, the delivery of services, and/or research activities. However, the review did not provide tools to support dialogue or practices for navigating complex (and often uncomfortable) issues of equity. It has been suggested that issues of power and equity are unavoidable in partnerships that are situated in contexts that are characterized by inequities.^44^ Principles aimed at guiding good partnering practices in global health, for example, emphasize the need to pay attention to how equity actions are integrated into the process of partnering itself.^45^^,^^46^ Equity-centric partnering pays attention to issues of equity as something **experienced** by people involved in partnerships, and therefore, requires attention to **how** equity is reflected both in the partnership overall and for each person involved in the partnership.

The EQT is unique and novel in its incorporation of evidence-informed practices for advancing equitable partnerships. It offers a reflective foundation to guide constructive dialogue about experiences of equity. The tool focuses on partnering practices that connect to equity experiences of individuals as well as experiences connected to the function of a partnership as a whole. Importantly, it is not intended to be used as a top-down set of standards or expectations for which people in positions of authority “collect” from others. It purposively does not include a score and ought to be used to support and inform constructive conversations rather than as a framework for evaluation. There may be particular contexts or additional considerations that people engaged in a GHP might want to reflect upon. For this reason, every section has space for additional statements to be added. This may include, for example, consideration of local or national contexts and potential donor obligations that influence equity-centered actions.

The unique and novel EQT should be used to support and inform constructive conversations on equity not as a framework for evaluation.

### Guide to Using the EQT

The EQT is a practical means of appreciating the quality of different aspects of a partnership in terms of established equity and promising practices. Each of its 4 domains of practice incorporates statements about an individual's experiences within the partnership: green, yellow, and red colors provide a visual cue for what GHPs might be invited to focus on in their reflection and dialogue about how their partnership is working. It is important for partners to discuss, as early as possible in the partnership, how the EQT will be used. Considerations might include the size of the partnership, the roles and responsibilities of all persons working in the partnership at different levels, and how results will be managed.

Conversations about equity create vulnerabilities and discomfort for many people, requiring facilitation skills and care. Across many disciplines, and even generally in public conversation, conversations about issues of equity are high risk. Everyone in a GHP experiences different positions of power. These experiences, the history of colonization, and ongoing neocolonial practices need to be confronted. Conversations about people's experiences of equity or inequity are welcomed in an inclusive and respectful way that attends to the cultural, emotional, physical, and career safety of all people who contribute. This might mean creating multiple tables of dialogue so that all people who should have a free and active voice can do so in a way that they feel safe. Partners can explore how to accomplish this together, designing an approach that works for them. There are excellent examples of workshops or training initiatives that focus on building awareness of, and responsiveness to, power dynamics, privilege, and equity that can be useful for GHPs that wish to embrace a consistent practice of equity-centric partnering.^16^^,^^46^

Because a partnership evolves over different phases, a periodic appreciation of equity and other considerations is appropriate at different times between initiation and completion. The EQT is intended to be used by partnering individuals or organizations who are initiating or are currently participating in a GHP. For this reason, the EQT is designed to be efficiently used as often and as strategically as needed to ensure adequate and timely reflection to guide responsiveness. It can also be used by individuals working at different levels within a partnership or by partner organizations as a whole (as represented by one or more of the lead partners). Issues of confidentiality should be discussed and agreed upon beforehand. The EQT can be completed either individually or collaboratively, or both, so that all voices can be heard. Ideally, an action plan should be established to implement recommended actions to mitigate indicators of concern. It needs to be reiterated that the primary intent of the EQT is to flag areas that need attention such that a conversation can follow, ultimately leading to improving the partnership. It doesn't necessarily matter if the tool is used to guide individual or group reflection—if there are areas where people's responses fall in the yellow or red zone or points where partners differ in their perception, the tool invites discussion about equity. The tool is intentionally not scored nor is it to be used to conclude that a partnership is, or is not, equitable. Organizations are encouraged to share their experiences with the EQT.

The EQT's primary intent is to flag areas that need attention such that a conversation can follow, ultimately leading to improving the partnership.

### Limitations

While the EQT benefited from input from different stakeholders during the online survey and workshops both in Canada and in 2 LMICs, there are limitations to its development. These include the use of strict inclusion criteria for the bibliographic search and a validation exercise limited to face and content validity. Field testing of the EQT for criterion validity across varied cultures and regions is needed. The value and uptake of the EQT in GHPs will only be able to be fully appreciated after it is used in varied types of partnerships and settings over time. Global health partnerships are encouraged to use EQT and are invited to share their learning experiences through commentary to GHPs.

## CONCLUSION

The EQT can support people involved in GHPs to advance equity in their actions and relationships, at all levels within the partnership. By engaging in a continuous process of learning and reflection, grounded in an intention of advancing equitable partnerships, GHPs can identify how their partnering can be more responsive and inclusive. By centering equity considerations in their processes, practices, and structure, GHPs can foster a dynamic and respectful culture of practicing equity in global health.

## Supplementary Material

21-00316-Larson-Supplement4.pdf

21-00316-Larson-Supplement5.pdf

21-00316-Larson-Supplement3.pdf

21-00316-Larson-Supplement2.pdf

21-00316-Larson-Supplement1.pdf

## Footnotes

* In July 2021, the CCGHR amalgamated with Canadian Society for International Health to become the Canadian Association for Global Health.

## Translation

**En Français**

**L'outil d'équité pour la valorisation des partenariats en santé mondiale**

**Résumé**

Les partenariats en santé mondiale impliquent des relations complexes entre des individus et des organisations, réunissant souvent des partenaires de pays à revenu élevé et de pays à revenu faible ou intermédiaire autour d'un travail effectué dans ces derniers. Les partenariats en santé mondiale s'inscrivent donc dans le contexte des inégalités mondiales et de leurs causes sociopolitiques et historiques sous-jacentes, telles que la colonisation. L'équité est un principe fondamental qui doit guider les partenariats en santé mondiale du début à la fin. Toutefois, la manière dont l'équité est intégrée et entretenue tout au long d'un partenariat reste un défi. Nous avons donc développé un outil simple d'utilisation permettant de valoriser un partenariat en santé mondiale tout au long de sa durée de vie, en portant une attention particulière à l'équité. L'élaboration de cet outil d'équité (l'EQT) s'est appuyée sur cinq éléments distincts: une revue exploratoire de la littérature scientifique publiée et évaluée par des pairs; une enquête en ligne et des entretiens téléphoniques de suivi; des ateliers au Canada, au Burkina Faso et au Vietnam; une synthèse interprétative critique; et un exercice de validation du contenu.

Les résultats suggèrent que les partenariats en santé mondiale génèrent des expériences d'équité ou d'iniquité, mais ne fournissent que peu de conseils sur la manière d'identifier et de répondre à ces expériences. L'EQT peut aider les personnes impliquées dans un partenariat à prendre en compte les implications en matière d'équité de toutes leurs actions, du début à la fin d'un partenariat, en passant par sa mise en œuvre et son achèvement. Lorsqu'il est utilisé pour guider un dialogue réfléchi avec l'intention claire de faire progresser l'équité dans et par le partenariat, cet outil offre une nouvelle approche de valorisation des partenariats en santé mondiale. Les professionnels de la santé mondiale, entre autres, peuvent appliquer l'EQT dans leurs partenariats pour apprendre ensemble comment cultiver l'équité dans leurs contextes uniques, au sein de ce qui devient une communauté de santé mondiale de plus en plus diversifiée, dynamique et réactive.

**Messages clés:**

- Il est nécessaire de faire progresser davantage l'équité dans les partenariats en santé mondiale.
- L'EQT présenté ici propose un guide pratique pour prendre en compte l'équité dans quatre domaines de pratique: gouvernance et processus, procédures et fonctionnement, progrès et impact, et enfin pouvoir et inclusion.
- L'EQT est axé sur l'équité, il est simple d'utilisation, peut soutenir un dialogue réfléchi à n'importe quel stade du partenariat et peut être utilisé par des personnes impliquées à tous les niveaux du partenariat.

**Principales implications:**

- L'EQT suscitera des questions qui inviteront chaque personne à marquer une pause pour réfléchir à son expérience au sein d'un partenariat.
- L'EQT permettra d'engager périodiquement un dialogue relationnel et réfléchi sur la manière dont l'équité est ressentie dans un partenariat en santé mondiale, donnant ainsi la possibilité aux partenaires de reconnaître, comprendre et faire progresser l'équité dans tous leurs processus.
- L'EQT invite à un dialogue réflexif sur la manière dont l'équité ou l'iniquité est vécue de différentes manières et à différents moments tout au long du processus de partenariat. Cet exercice nécessite de veiller à créer des conversations sûres, axées sur l'apprentissage, avec des intentions claires et le respect des contributions et de la vulnérabilité de toutes les personnes impliquées.

**Peer Reviewed**

**First published online:** April 19, 2022.
