# Supplementary material for: The Equity Tool for Valuing Global Health Partnerships
Source: Glob Health Sci Pract. 2022 Apr 28;10(2):e2100316. doi: 10.9745/GHSP-D-21-00316 (PMC9053142; doi:10.9745/GHSP-D-21-00316)
Supplement: 21-00316-Larson-Supplement4.pdf [file 21-00316-Larson-Supplement4.pdf]

**Supplement to:** Larson CP, Plamondon KM, Dubent L, et al. The Equity Tool for valuing global health partnerships. *Glob Health Sci Pract*. 2022;10(2): e2100316. https://doi.org/10.9745/GHSP-D-21-00316

# Supplément 4. Outil de valorisation des partenariats en santé mondiale axé sur l'équité (ÉQT)

**Gouvernance et processus :** Comment votre partenariat est composé, prend des décisions, et examine les contributions.

| *À propos de votre expérience* | Oui | Incertain | Non |
|---|---|---|---|
| 1. Les buts et objectifs de notre partenariat soutiennent mon propre apprentissage. | | | |
| 2. Je fais confiance aux autres dans notre partenariat. | | | |
| 3. Mon expertise technique et mes compétences sont connues et utilisées de manière significative. | | | |
| 4. Je soutiens les valeurs communes qui guident notre partenariat. | | | |
| 5. Le leadership est partagé de manière significative et appropriée. | | | |
| 6. Je participe aux décisions concernant la manière dont les fonds sont utilisés et gérés. | | | |
| 7. Je suis convaincu que les autres connaissent mes besoins et mes attentes. | | | |
| 8. Je ressens rarement une détresse éthique ou morale à propos de notre partenariat. | | | |
| *Ajoutez d'autres considérations spécifiques à votre situation* | | | |
| | | | |
| ***À propos du partenariat dans son ensemble*** | **Oui** | **Incertain** | **Non** |
| 9. Notre partenariat fixe des priorités qui répondent à ceux et celles qu'il cherche à servir. | | | |
| 10. Les buts et les objectifs de notre partenariat s'alignent sur les besoins et les priorités de la communauté. | | | |
| 11. Notre partenariat prend des décisions en toute transparence. | | | |
| 12. Notre partenariat permet un renforcement significatif des capacités et/ou un mentorat. | | | |
| 13. Notre partenariat garantit que les besoins et les attentes de tous les partenaires soient connus. | | | |
| 14. Notre partenariat implique la bonne combinaison de personnes. | | | |
| 15. Notre partenariat s'aligne de manière significative sur les besoins de chaque partenaire. | | | |
| 16. Notre partenariat respecte les obligations éthiques de tous les partenaires. | | | |
| *Ajoutez d'autres considérations spécifiques à votre situation* | | | |
| | | | |

**Procédures et opérations :** Comment votre partenariat fonctionne et met en œuvre des actions au jour le jour.

| *À propos de votre expérience* | Oui | Incertain | Non |
|---|---|---|---|
| 17. Je suis équitablement rémunéré.e pour mes contributions, qu'elles soient directes ou indirectes. | | | |
| 18. Mes contributions sont valorisées par les autres. | | | |
| 19. J'apprends des autres personnes impliquées dans notre partenariat. | | | |

| | | | |
|---|---|---|---|
| 20. J'ai confiance dans le fonctionnement quotidien de notre partenariat. | | | |
| 21. La communication est claire et cohérente. | | | |
| 22. On m'offre des possibilités d'améliorer mes compétences. | | | |
| *Ajoutez d'autres considérations spécifiques à votre situation* | | | |
| | | | |
| ***À propos du partenariat dans son ensemble*** | **Oui** | **Incertain** | **Non** |
| 23. Les décisions budgétaires impliquent tous les partenaires. | | | |
| 24. Notre partenariat est attentif à la viabilité financière. | | | |
| 25. Les ressources financières et autres sont équitablement partagées entre les partenaires. | | | |
| 26. Notre partenariat est attentif à son bon fonctionnement. | | | |
| 27. Notre partenariat s'efforce de tirer les leçons de l'expérience. | | | |
| 28. La compensation financière pour le personnel (salaires, indemnités journalières, etc.) est équitable. | | | |
| 29. Notre partenariat utilise des mécanismes efficaces de responsabilisation. | | | |
| 30. Nous travaillons activement à l'identification et à la résolution des problèmes éthiques. | | | |
| 31. Notre partenariat dispose de la bonne combinaison de compétences et de capacités. | | | |
| *Ajoutez d'autres considérations spécifiques à votre situation* | | | |
| | | | |

**Progrès et impacts :** La différence que votre partenariat fait pour les partenaires et ceux et celles qu'il sert.

| ***À propos de votre expérience*** | **Oui** | **Incertain** | **Non** |
|---|---|---|---|
| 32. Participer à ce partenariat est une expérience enrichissante. | | | |
| 33. Mes contributions sont reconnues par les autres. | | | |
| 34. Je suis d'accord avec les décisions concernant la propriété intellectuelle et la publication. | | | |
| 35. J'ai bon espoir quant aux avantages de notre partenariat. | | | |
| 36. Je me sens bien dans ma participation au partenariat. | | | |
| *Ajoutez d'autres considérations spécifiques à votre situation* | | | |
| | | | |
| ***À propos du partenariat dans son ensemble*** | **Oui** | **Incertain** | **Non** |
| 37. L'élaboration de produits d'application des connaissances répond aux besoins d'alphabétisation, à la culture et au contexte de ceux et celles que notre partenariat cherche à servir. | | | |
| 38. Notre partenariat s'efforce de bénéficier à des organisations, des communautés ou des groupes en dehors du partenariat lui-même. | | | |
| 39. Notre partenariat a un impact positif au niveau local. | | | |
| 40. Notre partenariat a un impact positif plus large. | | | |
| 41. Les questions de propriété intellectuelle et de publication sont discutées ouvertement. | | | |
| 42. La durabilité et la planification future sont ouvertement discutées. | | | |

| | | | |
|---|---|---|---|
| 43. Ce partenariat offre des opportunités aux étudiant.e.s et aux jeunes professionnel.le.s. | | | |
| *Ajoutez d'autres considérations spécifiques à votre situation* | | | |
| | | | |

**Pouvoir et inclusion** : Comment votre partenariat adopte activement la diversité et répond aux questions de pouvoir.

| ***À propos de votre expérience*** | **Oui** | **Incertain** | **Non** |
|---|---|---|---|
| 44. Ma contribution est importante dans notre partenariat. | | | |
| 45. J'ai la possibilité d'identifier et de partager mes propres attentes et besoins. | | | |
| 46. Ma contribution façonne ce qui est fait et la façon dont cela est fait. | | | |
| 47. Les autres membres de notre partenariat me respectent et respectent les contributions que j'apporte. | | | |
| 48. D'autres membres de notre partenariat respectent ma culture ainsi que mon savoir-être et mon savoir-faire. | | | |
| 49. Je suis convaincu.e que les personnes qui devraient être incluses dans notre partenariat le sont. | | | |
| *Ajoutez d'autres considérations spécifiques à votre situation* | | | |
| | | | |
| ***À propos du partenariat dans son ensemble*** | **Oui** | **Incertain** | **Non** |
| 50. Notre partenariat dispose d'un mécanisme efficace de résolution des conflits. | | | |
| 51. Mon organisation ou ma communauté est bien représentée dans ce partenariat. | | | |
| 52. Diverses perspectives sont représentées dans notre partenariat. | | | |
| 53. Notre partenariat est conscient et discute activement de la manière d'atténuer les inégalités inhérentes entre les partenaires. | | | |
| 54. Tous les partenaires sont appréciés et entendus, indépendamment de leur genre, de leur origine ethnique, de leurs capacités, de leur appartenance à un groupe ethnique, de leur classe, de leur éducation ou de toute autre position sociale. | | | |
| 55. La contribution de chaque partenaire est reconnue et appréciée de manière équitable. | | | |
| *Ajoutez d'autres considérations spécifiques à votre situation* | | | |
| | | | |
