# Supplementary material for: The Equity Tool for Valuing Global Health Partnerships
Source: Glob Health Sci Pract. 2022 Apr 28;10(2):e2100316. doi: 10.9745/GHSP-D-21-00316 (PMC9053142; doi:10.9745/GHSP-D-21-00316)
Supplement: 21-00316-Larson-Supplement5.pdf [file 21-00316-Larson-Supplement5.pdf]

Supplement to: Larson CP, Plamondon KM, Dubent L, et al. The Equity Tool for valuing global health partnerships. *Glob Health Sci Pract*. 2022;10(2): e2100316. https://doi.org/10.9745/GHSP-D-21-00316

Supplement 5. Recommended background references for essential topics in support of the application of the EQT

Principles of equity, power and privilege

Plamondon KM, Bisung E. The CCGHR principles for global health research: centering equity in research, knowledge translation, and practice. *Soc Sci Med* 2019;239, 112530.

Nixon S. The coin model of privilege and critical allyship: implications for health. BMC Public Health 2019;19:1637. https://doi.org/10.1186/s12889-7884-9

Core shared values among partners

WHO World Health Report 2003. Core values for global health partnership. https://www.who.int/whr/2003/overview/en/index2.html

Eichbaum QG, Adams LV, Evert J, *et al*. Decolonizing global health education: rethinking institutional partnership and approaches. *Acad Med* 2020. doi:10.1097/ACM.0000000000003473

CCGHR (Canadian Coalition for Global Health Research). Partnership Assessment Tool. Ottawa, Canada: CCGHR 2009. Accessed February 17, 2021 at https://www.ccghr.ca/resources/partnerships-and-networking/partnership-assessment-tool/

Human rights and sustainability

WHO. Human rights and health. 2017. https://www.who.int/news-room/fact-sheets/detail/human-rights-and-health

Gender equity guidelines

WHO. 10 key issues in ensuring gender equity in the global health workforce. 2019. https://www.who.int/news-room/feature-stories/detail/10-key-issues-in-ensuring-gender-equity-in-the-global-health-workforce

Government of Canada. Feminist International Assistance Gender Equality Toolkit for Projects. 2019. https://www.international.gc.ca/world-monde/funding-financement/gender_equality_toolkit-trousse_outils_egalite_genres.aspx?lang=eng
