# Supplementary material for: The Equity Tool for Valuing Global Health Partnerships
Source: Glob Health Sci Pract. 2022 Apr 28;10(2):e2100316. doi: 10.9745/GHSP-D-21-00316 (PMC9053142; doi:10.9745/GHSP-D-21-00316)
Supplement: 21-00316-Larson-Supplement3.pdf [file 21-00316-Larson-Supplement3.pdf]

Supplement to: Larson CP, Plamondon KM, Dubent L, et al. The Equity Tool for valuing global health partnerships. *Glob Health Sci Pract*. 2022;10(2): e2100316. https://doi.org/10.9745/GHSP-D-21-00316

Supplement 3. Definitions of Selected Words/Terms

Accountability: means being responsible for decisions and actions made: by oneself, by individual partners or by the partnership as a whole. There are many ways in which accountability can be ensured in a partnership and these should be specified from the outset of a partnership.

Authority: means having the power to make decisions. Within a partnership, authority is designated to one or more persons who will have decision-making power for specific activities (e.g. to use and allocate resources, to assign responsibilities, and so on).

Capacity building: Capacity building is the strengthening of competencies, at the individual, community, organization or system level. It can also occur in a bidirectional way.

Champion: A champion is an enthusiastic, committed and convincing promoter of the partnership, actively promoting the partnership in different audiences and in different fora. A champion can be a celebrity, a community leader, or other person who stands out in some way which can be of benefit to the partnership. A partnership can have more than one champion.

Commitment to the future: This relates to the principle of sustainability and the commitment to invest in a more equitable world where development and environmental issues, and human rights, including the right to health, are addressed.

Cultural competency: This refers to the ability to understand and value cultural differences, ways of thinking and doing, and to effectively communicate and interact with people from different cultures.

Ethical issues: The partnership's activities are guided by the universal ethical principles of respect for autonomy, beneficence and social justice. They inform reasons for acting, or refraining from acting, and for approving, or not approving, conduct within the partnership.

Financial accountability refers to accountability in the way partnership funds are used and managed.

Gender equality: Within the partnership there are equal numbers of women and men at all levels of participation. This also applies to hiring processes, assignment of tasks and performance evaluations. Only in rare circumstances, which should be made explicit, should one gender predominate over another (e.g. female interviewers administering a questionnaire on reproductive health to women).

Gender equity: Within the partnership there are opportunities for all genders to voice their opinion in a considered, fair, respectful and open manner. All genders can expect to fully participate within the partnership.

Governance: This includes the framework within which different levels of a partnership act with respect to how decisions are made, implemented, assessed and reported. Transparency and accountability are the cornerstones of good governance.

Knowledge translation: This is the communication, exchange, and application of knowledge by relevant stakeholders to ultimately improve people's health. It helps accelerate the benefits of global and local innovation in strengthening health systems.

Mutual respect: All partners and individuals in a partnership are equally respected and appreciated through mutual listening, understanding and respect of views and positions.

Ownership: Ownership means that each partner co-owns the outputs of the partnership. Ownership can be shared equally by all partners or all partners can agree on a specific, equitable designation of ownership for certain outputs.

Partner: Partners are individuals or groups of individuals (e.g. from government or non-governmental organizations, from universities or research institutions, from hospitals or communities, etc.) who collaborate together to achieve a common goal.

Supplement to: Larson CP, Plamondon KM, Dubent L, et al. The Equity Tool for valuing global health partnerships. *Glob Health Sci Pract*. 2022;10(2): e2100316. https://doi.org/10.9745/GHSP-D-21-00316

Partnership: A partnership consists of two or more organizations collaborating together to achieve a common goal.

Partnership agreement: A partnership agreement is a written contract between partners which sets out the terms and conditions of the relationship between the partners, including (but not limited to): distribution of funds and resources, partnership governance and management, duties of each partner and agreed upon procedures for changes or termination of the agreement.

Power relationships: Power relationships are recognized interactions among individuals which reflect differences in roles within a partnership and/or which stem from social differences between individuals in a partnership. Equitable partnerships recognize that power relationships exist or may develop within a partnership and have instituted effective and practical mitigation processes to avoid or manage conflicts.

Reciprocity: Reciprocity refers to the respective contributions made by all partners participating in a partnership. Each partner, or partner organization, is recognized as contributing to, and benefiting from, participation in the partnership.

Responsibilities: The tasks or duties that individuals participating in the partnership are expected to complete as a function of their respective roles.

Risk management: This refers to an on-going process of identification and assessment of events that could negatively impact the partnership (in terms of resources, timeline, processes, people, etc.), as well as the strategy developed to mitigate these events.

Roles: The functions of individuals within a partnership according to their designated position within the partnership.

Shared values: These are agreed upon principles which guide all actions and behaviors of the partnership, including those of each partner and individuals within each partner's organization. They may also be referred to as core values.

Stakeholders: A stakeholder is an individual, or an identifiable group of individuals or organizations, who have a vested interest in the activities of the partnership. Stakeholders may be governments, local authorities, communities, local organizations, etc.

Transparency: The open disclosure to all partners and, where appropriate, stakeholders, other persons in the partnership, governments, local authorities, communities, local organizations, etc., of decisions, activities, results and outputs of the partnership, including their respective underlying reasons and processes.

Valuation: Valuation refers to an appreciation of the value of some thing. Here, it is used in the context of considering (i.e. valuing) different aspects of equity within a global health partnership. This valuation can be done at different times during a partnership, and by different individuals within a partnership, with the intent of ensuring an equitable, best practice culture within the partnership.
