# Supplementary material for: The Equity Tool for Valuing Global Health Partnerships
Source: Glob Health Sci Pract. 2022 Apr 28;10(2):e2100316. doi: 10.9745/GHSP-D-21-00316 (PMC9053142; doi:10.9745/GHSP-D-21-00316)
Supplement: 21-00316-Larson-Supplement2.pdf [file 21-00316-Larson-Supplement2.pdf]

Supplement to: Larson CP, Plamondon KM, Dubent L, et al. The Equity Tool for valuing global health partnerships. *Glob Health Sci Pract*. 2022;10(2): e2100316. https://doi.org/10.9745/GHSP-D-21-00316

Supplement 2. Characteristics and Overview of Included Articles

CB=Capacity Building; SD=Sustainable Development; R=Research

First Author Year	GHP Type	Narrative Description of GHP(s) Assessed	Partnership Evaluation Framework Used	Primary Methodology (Methods)
Beran [1] 2016	CB-SD-R	Northern University partnering with Southern Health Systems, NGOs, professional associations, and universities	Selected elements of the Emerson (2011) Collaborative Governance Framework (CITE)	Retrospective analysis using a collaborative governance framework
Birch [2] 2013	CB-R	A North-South research and clinical partnership in a graduate nursing program	Mercer (2008) tool for assessing participatory research projects	Mixed methods (literature review + case study using interviews)
Bruen [3] 2014	SD	Multilateral public-private partnership with Northern 'funders' and Southern 'recipients'; multi-stakeholder board	None	In-depth discussion of the concept of partnership accountability
Buse [4] 2011	SD	Multi-lateral partnerships, including: health product access; health product development; or global coordination/ financing mechanisms	None	Meta-Synthesis of 8 public-domain evaluations of GHPs
Citrin [5] 2017	CB-R	Northern university partnering with Southern Health System and NGO	Tropical Health & Education Trust Principles of Partnership Framework	Review (methods unclear)
Coffey [6] 2018	CB-SD	Healthcare delivery partnership among pharmaceutical companies, academia, International NGOs, UN agencies, and bilateral funders, with implementation in Global South	Brown's Coalition Functioning	Mixed methods case study
Dean [7] 2015	CB-R	Northern University partnering with Southern Universities, with funding scheme to facilitate research capacity building and exchange between UK and African universities	None	Mixed methods retrospective evaluation (cross-sectional survey + semi-structured interviews + focus groups)

Supplement to: Larson CP, Plamondon KM, Dubent L, et al. The Equity Tool for valuing global health partnerships. *Glob Health Sci Pract*. 2022;10(2): e2100316. https://doi.org/10.9745/GHSP-D-21-00316

First Author Year	GHP Type	Narrative Description of GHP(s) Assessed	Partnership Evaluation Framework Used	Primary Methodology (Methods)
El-Bchearaoui [8] 2017	SD	Results-based aid partnership involving philanthropic agencies, international financial institutions, international NGOs, Southern health systems and health professionals, with a topical focus on maternal-child health	PARTNER tool	Grounded theory process evaluation (document review + key informant interviews + focus groups + partnership analysis)
Herrick [9] 2018	CB-SD	Northern university and health systems network partnering with Southern university and health systems network	None	Ethnographic research (methods unclear)
John [10] 2016	R	Northern universities partnering generally with Southern partners for research purposes	None	Reflective questions asked open-ended questions
Kamya [11] 2017	R	Public private partnership for health interventions implementation in Global South, with topical focus on vaccines	Binkerhoff's Assessing and Improving Partnership Relationships and Outcomes framework	Mixed-methods case study (document review + in-depth interviews + social network analysis)
Leffers [12] 2011	CB-SD	Northern academic and professional nurses going to Southern countries for research, education and electives, or practice (i.e., missions)	Developed a framework	Grounded theory
Lipsky 2016 [13]	SD	Northern development agencies partnering with Southern health systems and NGOs, with topical focus on family planning	Binkerhoff's Assessing and Improving Partnership Relationships and Outcomes framework	Evaluation
Murphy [14] 2015	R	Northern researchers partnering with Southern researchers and research users, generally	Develop a tool	Sequential consultation, using 3-day regional workshops in Africa, South Asia, and Latin America + pre- and post- workshop engagement

Supplement to: Larson CP, Plamondon KM, Dubent L, et al. The Equity Tool for valuing global health partnerships. *Glob Health Sci Pract*. 2022;10(2): e2100316. https://doi.org/10.9745/GHSP-D-21-00316

First Author Year	GHP Type	Narrative Description of GHP(s) Assessed	Partnership Evaluation Framework Used	Primary Methodology (Methods)
Ndenga [15] 2016	CB	Northern universities partnering with Southern universities and health systems, with a topical focus on teaching	None	Online survey, regression analysis
Neuhann [16] 2017	SD-R	Northern funding agency and universities partnering with Southern hospitals, with topical focus on health professional capacity	Capacity WORKS model	Document Analysis
Njelesani [17] 2013	R	Northern university and researchers partnering with Southern researchers & NGO, with topical focus on applied health sciences	Global Health Research Initiative Framework (Boutillier)	Retrospective partnership evaluation
Pattberg[18] 2016	SD	Multi-stakeholder partnerships for 'sustainable development', generally	Develop nine considerations	Unclear ("findings were distilled from the literature" and involved consultation with many major international organizations)
Perez-Escamilla [19] 2014	SD-R	School-based public-private partnerships led by Northern university, with implementation in Global South	Alexander 12 Principles	Qualitative analysis of documents and key informant interviews
Ramaswamy [20] 2016	SD	Northern university partnering with international NGO, located in multiple Global South countries, with topical focus on clinical partnerships, training, and development	Kybale Model (internally developed)	Case study (document analysis + focus groups + interviews + clinical outcomes data) (unclear methodology)
Ridde [21] 2011	R	Northern universities partnering with Southern universities, generally	None	Exploratory qualitative research (focus groups)
Sandwell [22] 2018	CB-SD	North-South professional associations and universities twinning partnership	None	N/A
Sriharan [23] 2016	CB-SD	Northern universities partnered with Southern hospitals, with topical focus on health professional continuing education	Develop theory	2-part Realist Approach: Realist review + Interviews
Steenhoff [24] 2017	CB-SD	Northern academics and health professionals engaged	None	Review + expert panel

Supplement to: Larson CP, Plamondon KM, Dubent L, et al. The Equity Tool for valuing global health partnerships. *Glob Health Sci Pract*. 2022;10(2): e2100316. https://doi.org/10.9745/GHSP-D-21-00316

First Author Year	GHP Type	Narrative Description of GHP(s) Assessed	Partnership Evaluation Framework Used	Primary Methodology (Methods)
		in collaborative initiatives with Global South, generally, with topical focus on pediatrics		
Storr [25] 2018	CB-R	Northern universities partnering with Southern countries to provide "ethical and sustainable fieldwork experiences" (p. 34) of Northern students	Working Group for Ethical Guidelines on Global Health Training (WEIGHT) guidelines	Quality improvement (unclear methodology)
Theissen [26] 2018	CB-SD	Public–private partnership between Southern health system and Northern government and industry, with topical focus on maternal health	None	Qualitative methodology (interviews + focus groups)
Underwood [27] 2016	CB	Northern university partnering with Southern NGOs to provide Northern midwifery and nursing students community-based learning placements	Leffers & Mitchell Framework for Partnership and Sustainability in Global Health	Descriptive exploratory qualitative methods (narrative data)
Upvall [28] 2018	CB-SD	Southern perspectives on North-South partnerships to provide Northern nursing students international electives in Southern settings	Leffers & Mitchell Framework for Partnership and Sustainability in Global Health	Grounded theory
Yarmoshuk [29] 2018	CB	Northern universities partnering with Southern universities, with a topical focus on capacity building of health professionals and health professional programs	Kernaghan's classification of partnerships	Mixed methods (quantitative analysis of 125 distinct partnerships; qualitative analysis of characteristics contributing to partnership's value)
Yassi [30] 2014	CB-R	Northern universities partnering with Global South health professionals, with topical focus on interventions for infectious disease	Medical Research Council Campbell et al. frameworks for design and evaluation of complex interventions for health	Narrative qualitative approach

1 Beran D, Aebischer Perone S, Alcoba G, *et al*. Partnerships in global health and collaborative governance: lessons learnt from the Division of Tropical and Humanitarian Medicine at the Geneva University Hospitals. *Globalization Health* 2016;12(1):14, doi:10.1186/12992-016-0156-x

2 Birch AP, Tuck J, Malata A, *et al*. Assessing global partnerships in graduate nursing. *Nurs Educ Today* 2013;33(11):1288–94, doi:10.1016/j.nedt.2013.03.014

3 Bruen C, Brugha R, Kageni A, *et al*. A concept in flux: Questioning accountability in the context of global health cooperation. *Globalization Health* 2014;10:73 doi:10.1186/s 12992-014-0073-9,

4 Buse K, Tanaka S. Global public-private health partnerships: lessons learned from ten years of experience and evaluation. *Int Dent J* 2011;61(Suppl 2):2–10 doi:10.1111/j.1875-595x.2011.00034.x

5 Citrin D, Mehanni S, Acharya B, *et al*. Power, potential, and pitfalls in global health academic partnerships: review and reflections on an approach in Nepal. *Global Health Action* 2017;10(1):1367161 doi:10.1080/16549716.2017.1367161

6 Coffey PS, Hodgins S, Bishop A. Effective collaboration for scaling up health technologies: A case study of the chlorhexidine for umbilical cordcare experience. *Glob Health Sci Pract* 2018;6(1):178–91.

7 Dean L, Njelesani J, Smith H, *et al*. Promoting sustainable research partnerships: A mixed-method evaluation of a United Kingdom-Africa capacity strengthening award scheme. *Health Res Policy Syst* 2015;13:1–10 doi:10.1186/s 12961-015-0071-2

8 El Bcheraoui C, Palmisano EB, Dansereau E, *et al*. Healthy competition drives success in results-based aid: Lessons from the Salud Mesoamérica Initiative. *PLoS One* 2017;12(10):e0187107 doi:10.1371/journal.pone.0187107

9 Herrick C, Brooks A. The binds of global health partnership: Working out working together in Sierra Leone. *Med Anthropol Q* 2018;32(4):520–38 doi:10.1111/maq.12462

10 John CC, Ayodo G, Musoke P. Successful Global Health Research Partnerships: What Makes Them Work? *Am J Trop Med Hyg* 2016;94(1):5–7 doi:10.4269/ajtmh.15-0611.

11 Kamya C, Shearer J, Asiimwe G, Carnahan E, Salisbury N, Waiswa P, et al. Evaluating global health partnerships: A case study of a Gavi HPV vaccine application process in Uganda. Int J Heal Policy Manag. 2016;6(6):327–38.

12 Leffers J, Mitchell E. Conceptual model for partnership and sustainability in global health. Public Health Nurs. 2011;28(1):91–102.

13 Lipsky AB, Gribble JN, Cahaelen L, Sharma S. Partnerships for policy development: A case study from Uganda’s Costed implementation Plan for Family Planning. Glob Heal Sci Pract. 2016;4(2):284–99.

14 Murphy J, Hatfield J, Afsana A, et al. Making a commitment to ethics in global health research partnerships: a practical tool to support ethical practice. *J Bioethic Inq* 2015;12:137-146.

15 Ndenga E, Uwizeye G, Thomson DR, Uwitonze E, Mubiligi J, Hedt-Gauthier BL, et al. Assessing the twinning model in the Rwandan Human Resources for Health Program: Goal setting, satisfaction and perceived skill transfer. Glob Heal. 2016;12:4.

16 Neuhann F, Barteit S. Lessons learnt from the MAGNET Malawian-German Hospital Partnership: the German perspective on contributions to patient care and capacity development. Glob Heal. 2017;13(1):50.

17 Njelesani J, Stevens M, Cleaver S, Mwambwa L, Nixon S. International Research

Partnerships in Occupational Therapy: A Canadian-Zambian Case Study. Occup Ther Int. 2013;20(2):75–84.

18 Pattberg P, Widerberg O. Transnational multistakeholder partnerships for sustainable development: Conditions for success. *Ambio* 2016;45(1):42–51 doi:10.1007/s13280-015-0684-2

19 Perez-Escamilla R. Innovative healthy lifestyles school-based public-private partnerships designed to curb the childhood obesity epidemic globally: Lessons learned from the Mondelez International Foundation. *Food Nutr Bull* 2018;39(1S):S3–21 doi:10.1177/0379572118767690

20 Ramaswamy R, Kallam B, Kopic D, *et al*. Global health partnerships: Building multi-national collaborations to achieve lasting improvements in maternal and neonatal health. *Globalization Health* 2016;12(1):22 doi:10.1186/s12992-016-0159-7

21 Ridde V, Capelle F. La recherche en santé mondiales et les défis des partenariats Nord-Sud. *Can J Public Health* 2011;102(2):152–6.

22 Sandwell R, Bonser D, Hebert E, *et al*. Stronger together: midwiery twinning between Tanzania and Canada. *Globalization Health* 2018;14:123 doi:10.1186/s12992-018-0442-x

23 Sriharan A, Harris J, Davis D, *et al*. Global health partnerships for continuing medical education: Lessons from successful partnerships. *Health Sys Reform* 2016;2(3):241-253.

24 Steenhoff AP, Crouse HL, Lukolyo H, *et al*. Partnerships for Global Child Health. *Pediatrics* 2017;140(4):10.

25 Storr C, MacLachlan J, Krishna D, *et al*. Building sustainable fieldwork partnerships between Canada and India: Finding common goals through evaluation. *World Fed Occup Ther Bull* 2018;74(1):34–43 doi:10.1080/14473828.2018.1432312

26 Thiessen J, Bagoi A, Homer C, *et al*. Qualitative evaluation of a public private partnership for reproductive health training in Papua New Guinea. *Rural Remote Health* 2018;18:4608 doi:10.22605/RRH4608

27 Underwood M, Gleeson J, Konnert C, *et al*. Global host partner perspectives: Utilizing a conceptual model to strengthen collaboration with host partners for international nursing student placements. *Public Health Nurs* 2016;33(4):351–9 doi:10.1111/phn.12258

28 Upvall MJ, Leffers JM. Revising a conceptual model of partnership and sustainability in global health. *Public Health Nurs* 2018;35(3):228–37 doi:10.1111/phn.12396

29 Yarmoshuk AN, Guantai AN, Mwangu M, *et al*. What makes international global health university partnerships higher-value? An examination of partnership types and activities fovoured at four East African universities. *Ann Glob Health* 2018;84(1):139-50 doi:10.29024/aogh.20

30 Yassi A, O'Hara LM, Engelbrecht MC, *et al*. Considerations for preparing a randomized population health intervention trial: lessons from a South African-Canadian partnership to improve the health of health workers. Glob Health Action 2014;7:23594 doi:10.3402/gha.v7.23594
