# Supplementary material for: The Equity Tool for Valuing Global Health Partnerships
Source: Glob Health Sci Pract. 2022 Apr 28;10(2):e2100316. doi: 10.9745/GHSP-D-21-00316 (PMC9053142; doi:10.9745/GHSP-D-21-00316)
Supplement: 21-00316-Larson-Supplement1.pdf [file 21-00316-Larson-Supplement1.pdf]

Supplement to: Larson CP, Plamondon KM, Dubent L, et al. The Equity Tool for valuing global health partnerships. *Glob Health Sci Pract*. 2022;10(2): e2100316. https://doi.org/10.9745/GHSP-D-21-00316

Supplement 1. Description of the Five Inputs Informing the Development of the EQT

Inputs	Description
Scoping Review	The objective of the scoping review was to gather indicators previously used in evaluating or assessing a GHP in ways that identify, consider, mitigate, or foster equity. A bibliographic search of the published peer-reviewed literature was conducted, with the following inclusion criteria: partnerships involved at least one partner from an HIC and one from an LMIC, where the partnership itself, rather than one or more projects undertaken by the partnership, had been the subject of an evaluation or assessment. Government-to-government partnerships were excluded. Any type of GHP was eligible, including those focused on research, capacity-building or sustainable development. Searches for articles published in English or French between 1 January 2010 and 16 February 2019 were carried out in Medline, Embase, CINAHL, Web of Science, and Scopus (for search terms in the title, abstract and keywords fields) and PsycINFO (in keywords only). Search terms included: assessment, evaluation; LMICs (based on the World Bank list); partnerships; characteristics (of a quality partnership); and global health.<br><br>A two-step screening process was initiated where all records identified by the search were initially screened by one reviewer (LD) using a liberal approach of inclusion of publications, and subsequently, by two additional reviewers (CPL and TWG), using a more conservative approach. This was followed by a full-text review of selected articles. Where discrepancies in selection of records occurred, a third reviewer (KMP) provided the final decision. The reviews were managed using the Covidence software program (www.covidence.org; Melbourne, Australia). Bibliographies of included articles were hand-searched as a secondary means of identifying additional relevant literature.
On-line survey and follow-up telephone interviews	Using LimeSurvey software, an on-line questionnaire-based survey, accessible in either English or French, was active between February 18 and April 15, 2019. The survey invited participants to share their experience of having participated in a GHP. It also asked about their experience in participating in assessing a GHP and were invited to share any tools they had used or developed. Canadian and international members of the CCGHR and the Regroupement stratégique en santé mondiale of the Réseau de recherche en santé des populations du Québec (a network of global health researchers in Québec, Canada) received a personal email invitation to participate in the survey. Participants in the on-line survey were also invited to take part in a telephone interview to further explore details of their partnership experience, including, if applicable, how the assessment informed the future activities of the partnership. Semi-structured interviews were conducted in either English

Supplement to: Larson CP, Plamondon KM, Dubent L, et al. The Equity Tool for valuing global health partnerships. *Glob Health Sci Pract*. 2022;10(2): e2100316. https://doi.org/10.9745/GHSP-D-21-00316

**Inputs**	**Description**
	or French using a standard template which could be expanded according to the responses provided.
Critical interpretive synthesis	CIS involves in-depth qualitative analysis of a complex and amorphous set of literature, informed by critical theory [16]. This synthesis involved all articles identified in the scoping review, and drew upon the CCGHR Principles for Global Health Research [14] and other critical scholarship in the field [17-19] to pose equity-centred questions about how equity considerations were reflected in the portrayal, justification, assessment, and description of GHPs [20]. This in-depth analysis identified the kinds of practices and processes within GHPs where equity can be advanced (or not). Results were then compared with those of the SR, interviews and workshops, cross-referenced and assessed against the CCGHR Principles for Global Health Research and tools or frameworks identified by participants during interviews, to develop content for a draft EQT.
Workshops	Two 1-day Canada-based (Montréal and Ottawa) and two 2-day internationally based (Hanoi, Vietnam and Ouagadougou, Burkina Faso) workshops were conducted, two in English and two in French. Findings of the scoping review were presented followed by additional discussion and context to identify which indicators were considered feasible and a priority, and whether the tool should be modified in line with partnership phases (start-up, implementation, completion).
Validation Exercise	The draft tool was reviewed by the research team and partners in Vietnam and Burkina Faso. Researchers and practitioners whose work contributed to other tools and guides for supporting GHPs were consulted and invited to provide feedback. It was also distributed to members of the Canadian Partnership for Women's and Children's Health (CanWaCH) - Health Metrics Working Group. Each consultation provided an opportunity to test the tool for face validity, feasibility, and clarity, generating minor revisions to clarify language and refine some functional element of the tool.
